# Further investigation of risk elements content in the bones of wild rodents from a polluted area in Slovakia

**DOI:** 10.1186/s13028-015-0138-7

**Published:** 2015-08-25

**Authors:** Monika Martiniakova, Radoslav Omelka, Birgit Grosskopf, Hana Duranova, Robert Stawarz, Ivan Balaz

**Affiliations:** Department of Zoology and Anthropology, Constantine the Philosopher University, 949 74 Nitra, Slovakia; Department of Botany and Genetics, Constantine the Philosopher University, 949 74 Nitra, Slovakia; Institute of Zoology and Anthropology, Georg-August University, 37 073 Göttingen, Germany; Institute of Biology, Krakow Pedagogical University, 31 054 Kraków, Poland; Department of Ecology and Environmentalistics, Constantine the Philosopher University, 949 01 Nitra, Slovakia

**Keywords:** Risk elements, Bone, Wild rodents, Slovakia

## Abstract

**Background:**

Wild rodents are suitable for monitoring environmental pollution and exposure risk assessment for people living in contaminated areas. The content of selected risk elements in the femora of bank vole (*Myodes glareolus*), yellow-necked mouse (*Apodemus flavicollis*) and wood mouse (*Apodemus sylvaticus*) was estimated from the Kolíňany area of Slovakia, which is characterized by a high degree of environmental pollution. The rodents were trapped in February 2011 using standard theriological methods. All animals (n = 32) were adult males in good physical condition. The concentrations of Fe, Cu, Zn and Ni in their bones were determined by atomic absorption spectrophotometry.

**Results:**

The highest concentrations of Fe and Cu were detected in the bones of yellow-necked mouse. Significant differences were observed for the content of Fe between *A. flavicollis* and *M. glareolus* (*P* < 0.05). The highest levels of Zn and Ni were found in the femora of wood mouse; however, significant differences were not detected between the rodents. Moreover, the concentrations of Cu, Zn and Ni were significantly higher in the bones of all three species (*P* < 0.05) in comparison with the values obtained in the same animal species at the same site in February 2007.

**Conclusions:**

Our results demonstrate an increased accumulation of Cu, Zn and Ni in the femora of *M. glareolus, A. flavicollis* and *A. sylvaticus* from the Kolíňany area and thus indicate towards ongoing contamination of this locality.

## Background

The importance of monitoring and studying the effect of various risk elements on living organisms has become critical in the last few decades; specifically in East and Central Europe. There are a number of ecological studies that have demonstrated that rodents are essential canary-type species useful in monitoring pollutant issues for their habitats. Rodents are the sentinels of man-made environmental pollution crises.

Various studies of wild rodents have revealed that they are able to accumulate a wide spectrum of pollutants which are present in the ecosystem [1, 2]. In addition, the pattern of risk elements distribution and their levels in various tissues of the rodents are similar to those found in humans [3, 4]. This makes rodents ideal for monitoring environmental pollution, as well as for evaluating the exposure risk for people living in a contaminated area [4, 5].

The bank vole (*Myodes glareolus,* formerly *Clethrionomys glareolus*, Schreber, 1780) is a small microtine rodent that is common throughout Europe and it is one of the most common woodland rodents in Slovakia. Seeds, fruits and green vegetation constitute about 44 % of their food, insects, earthworms and other invertebrates between 9 and 23 %, depending on the season, and in winter they add tree bark to their food [6, 7]. This species has been used to monitor environmental pollution from a variety of technogenic sources up to date [8, 9]. Mice from the genus *Apodemus* have been shown to be relevant pollution bioindicators [10–13]. The yellow-necked mouse (*Apodemus flavicollis*) and wood mouse (*Apodemus sylvaticus*) belong to the most dominant rodent species in Slovakia. The yellow-necked mouse is slightly larger and more brightly colored than the wood mouse. It eats mainly seeds, especially acorns, beech mast and hazel nuts, but it also consumes insects and other invertebrate as food [14]. The diet of the wood mouse consists of roots, grains, seeds, berries, nuts, grasses, grain kernels, fruits and insects [15].

Since bone can serve as a good biomarker of long-term accumulation of various risk elements including non-essential and essential metals, we analysed concentrations of selected essential metals (Fe, Cu, Zn, and Ni) in the femora of the three rodent species mentioned above. These metals are necessary for proper functioning of living organisms. The uptake and distribution of these biologically essential metals is physiologically regulated, in contrast to other non-essential elements [16]. However, essential metals can also produce toxic effects when their intake reaches high concentrations [17].

In our study, all wild rodents were trapped from the Kolíňany area of Slovakia which is considered as a heavily polluted region. Our earlier experiments focused on the determination of various risk elements in the bones of *M. glareolus, A. flavicollis* and *A. sylvaticus* in February 2007 and the results demonstrated significantly higher concentrations of Fe, Cu and Zn in the bones of bank voles from the Kolíňany area as compared to those from the Nováky area of Slovakia [18]. The Nováky region of Slovakia is generally considered to be strongly polluted region in Slovakia because of a localization of many sources of environmental contamination, e.g. Nováky chemical plant, Coal power station in Nováky, Handlová–Cígeľ mines.

Furthermore, a significantly higher content of Ni and Zn was found in the femora of yellow-necked mice and wood mice from the Kolíňany locality in comparison with the Nováky area [19]. Therefore, in addition to the determination of risk elements in the bones of wild rodents, we compared the present results with those obtained in the year 2007 [18, 19].

## Methods

**Fig. 1:**
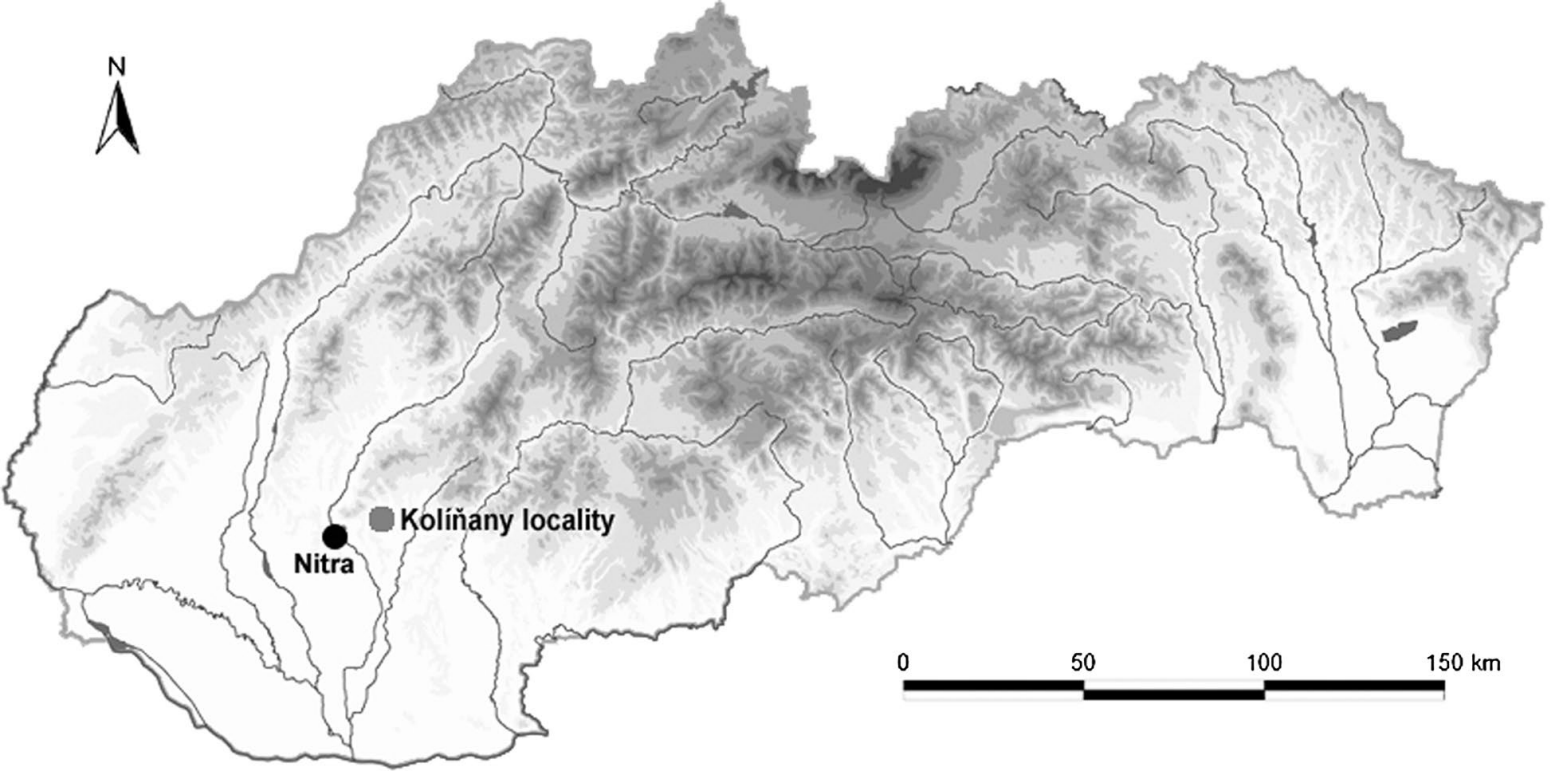
Map of investigated locality. The figure shows a location of investigated Kolíňany locality (GPS coordinates: N48°21′18.28″, E18°12′36.03″) which is situated near the Nitra city

The individuals of bank vole (*M. glareolus*, n = 14), yellow-necked mouse (*A. flavicollis*, n = 6) and wood mouse (*A. sylvaticus*, n = 12) were obtained by means of the standard theriological methods and procedures from wood ecosystems [20] in February 2011. The wild rodents were trapped near the water pond in Kolíňany (Nitra district, Slovakia; Fig. 1) which is located ~10 km away from the town Nitra and it is considered to be a heavily polluted region [18, 19]. Possible sources of pollution for this district are small factories, the application of agricultural chemicals, traffic pollution and the waste from large local industrial complexes. All animals caught were adult males (aged 4–5 months of age as determined by dental wear). They appeared to be in good physical condition and without gross lesions at necropsy. All procedures were approved by the Ministry of Environment of the Slovak Republic.

The concentrations of selected risk elements (Fe, Cu, Zn, and Ni) were estimated in both the femora of all the investigated rodents (n = 64) using the method of atomic absorption spectrophotometry (Perkin Elmer 4100 ZL) in a graphite furnace [21]. The tissue samples were kept at −18 °C until analysis. In the laboratory, the samples were dried at 105 °C until dry mass was obtained. Then, the bones were weighed (minimum 2 g) and digested in concentrated nitric acid at 90 °C for 5 h. Prior to analysis, the samples were diluted to 10 ml with distilled water. All metal concentrations were expressed on a dry weight basis in mg kg^−1^.

From the final data, basic statistical characteristics were calculated (mean, standard deviation, minimum, maximum, median). The analysis of variance and Scheffe test were used for comparison of risk elements content between species. The *T* test was applied to compare the present data with those obtained in February 2007 [18, 19].

## Results and discussion

The concentrations of selected risk elements (Fe, Cu, Zn, and Ni) in the femora of *M. glareolus*, *A. flavicollis* and *A. sylvaticus* from the Kolíňany area are listed in Table 1. The highest concentrations of Fe and Cu were detected in the bones of yellow-necked mice. Significant differences were observed for the content of Fe between *A. flavicollis* and *M. glareolus* (*P* < 0.05). The highest levels of Zn and Ni were found in the femora of wood mice; however, significant differences were not detected between the rodents.

**Table 1:** Concentrations of risk elements in the femora of wild rodents from the Kolíňany area

Species	Fe (mg kg^−1^)	Cu (mg kg^−1^)	Zn (mg kg^−1^)	Ni (mg kg^−1^)
*Myodes glareolus* (1)				
x	197.26	62.63	241.73	26.94
sd	67.36	19.45	19.28	5.59
min	123.24	42.08	217.74	19.26
max	286.58	88.79	268.94	32.06
med	197.92	61.8	245.03	29.21
*Apodemus flavicollis* (2)				
x	240.28	69.94	239.94	29.58
sd	75.14	20.03	52.61	9.87
min	154.46	61.75	187.39	13.71
max	330.89	109.23	316.46	45.83
med	277.91	85.62	272.34	26.98
*Apodemus sylvaticus* (3)				
x	215.46	68.78	244.74	30.79
sd	44.78	13.58	46.58	10.21
min	171.68	52.82	196.79	9.32
max	277.32	85.34	294.54	58.09
med	206.41	68.49	243.81	27.87
Scheffe test	1:2 (+)	NS	NS	NS

*x* Mean, *sd* standard deviation,* min* minimum,* max* maximum,* med* median, + *P* < 0.05,* NS* non-significant changes

We observed a higher concentration of Zn in the femora of *M. glareolus* in comparison with the data reported by Milton et al. [8]. These investigators analysed Zn concentration (173 ± 5.1 μg g^−1^ dry weight) in the femora of bank voles trapped at the contaminated, unused Pb mine at Frongoch in west Wales. In contrast, Zn concentration in the bones of bank voles from the Kolíňany area was lower than the value (261.1 ± 7.4 μg g^−1^ dry weight) reported by Milton and Johnson [22], who analysed femora of laboratory-bred bank voles exposed to increased levels of dietary Zn.

Also, higher concentrations of Zn and Fe were detected in the femora of *A. flavicollis* in our study as compared to those observed by Damek-Poprawa and Sawicka-Kapusta [4]. These authors determined the content of Zn and Fe in the femur of yellow-necked mice from Zn smelters in Bukowno (Poland) which are considered to be extremely polluted (Zn concentration 166.3 ± 7.6 μg g^−1^ dry weight, Fe concentration 153.0 ± 9.9 μg g^−1^ dry weight).

It is interesting to note that the concentrations of Cu, Zn and Ni were significantly higher in the bones of all three wild rodents in our study in comparison with the values obtained in the same animal species at the same site (Kolíňany) in February 2007 (Table 2). Therefore, our results demonstrate the increased accumulation of these elements in the femora of the rodents investigated and thus indicate towards the ongoing contamination of this locality. This fact can be explained by intensive agricultural production and subsequent contamination of the soil, water, and food, by traffic pollution, as well as by various factories and industrial zones in western Slovakia. These factors are present today and they were also problematic in the recent past (e.g. production of Ni in Sereď and its dumping sites) [23].

**Table 2:** Comparison of risk elements content with data obtained by Martiniaková et al. [18, 19]

Species/study	Fe (mg kg^−1^)	Cu (mg kg^−1^)	Zn (mg kg^−1^)	Ni (mg kg^−1^)
*Myodes glareolus*—present study				
x	197.26	62.63	241.73	26.94
sd	67.36	19.45	19.28	5.59
*Myodes glareolus*—study of Martiniaková et al. [18]				
x	212.99	4.16	188.55	9.52
sd	52.27	2.1	21.61	2.8
T-test	NS	+	+	+
*Apodemus flavicollis*—present study				
x	240.28	69.94	239.94	29.58
sd	75.14	20.03	52.61	9.87
*Apodemus flavicollis*—study of Martiniaková et al. [19]				
x	163.27	4.43	143.84	9.16
sd	73.91	1.19	16.52	1.89
T-test	+	+	+	+
*Apodemus sylvaticus*—present study				
x	215.46	68.79	244.74	30.79
sd	44.78	13.58	46.58	20.21
*Apodemus sylvaticus*—study of Martiniaková et al. [19]				
x	109.1	3.33	147.55	7.8
sd	35.61	1.06	13.35	0.84
T-test	+	+	+	+

*x* mean,* sd* standard deviation, + *P* < 0.05,* NS* non-significant changes

In general, the intensive agricultural production and the use of agrochemicals are characteristic for the whole region of Nitra. It is known that the application of agrochemicals can lead to a higher accumulation of specific elements, including Ni, Cu and Zn into the soil [24, 25]. In addition, there is heavy road traffic near the capture locality, which is also considered to be a significant source of risk elements that has a potential to be transported by air flow over large distances. According to Blagojevic et al. [16], at least 90 % of the metals in road runoff consist of Cu, Zn and Pb. There is also a possibility of falling dust being transported in the air from large industrial regions, such as Bratislava, Vienna, Budapest, or factories near the Nitra district. This hypothesis may be supported by a study indicating the possibility of the long range transportation of various xenobiotics [26].

Since mechanisms of heavy metals bioaccumulation are very similar in mammals whatever area they occupy [16], our results could also be extrapolated on humans living in the Kolíňany area.

## Conclusions

The accumulation of selected risk elements (Fe, Cu, Zn, and Ni) in the femora of bank vole, yellow-necked mouse and wood mouse from the Kolíňany area was investigated in the present study. The highest concentrations of Fe and Cu were detected in the bones of yellow-necked mouse. Significant differences were observed for the concentration of Fe between yellow-necked mouse and bank vole (*P* < 0.05). The highest levels of Zn and Ni were found in the femora of wood mouse; however, significant differences were not detected between the rodents. The concentrations of Cu, Zn and Ni were significantly higher in the bones of all three species in our study in comparison with the values obtained in the same animal species at the same site in the year 2007. This study finds a need for a continuation of the monitoring of heavy metal levels in Central Europe environments, specifically in the Nitra district.
